# Complete Genome Sequences of African Salmonella enterica Serovar Enteritidis Clinical Isolates Associated with Bloodstream Infection

**DOI:** 10.1128/MRA.01452-20

**Published:** 2021-03-25

**Authors:** Blanca M. Perez-Sepulveda, Alexander V. Predeus, Wai Yee Fong, Christopher M. Parry, John Cheesbrough, Paul Wigley, Nicholas A. Feasey, Jay C. D. Hinton

**Affiliations:** a Institute of Infection, Veterinary & Ecological Sciences, University of Liverpool, Liverpool, United Kingdom; b Institute of Infection and Global Health, University of Liverpool, Liverpool, United Kingdom; c Liverpool School of Tropical Medicine, Liverpool, United Kingdom; d Malawi-Liverpool-Wellcome Programme, Blantyre, Malawi; University of Maryland School of Medicine

## Abstract

We report the complete genome sequencing and annotation of four Salmonella enterica serovar Enteritidis isolates, two that are representative of the Central/Eastern African clade (CP255 and D7795) and two of the Global Epidemic clade (A1636 and P125109).

## Abstract (precis)

We report the complete genome sequencing and annotation of four Salmonella enterica serovar Enteritidis isolates, two that are representative of the Central/Eastern African clade (CP255 and D7795) and two from the Global Epidemic clade (A1636 and P125109).

## ANNOUNCEMENT

Salmonella enterica serovar Enteritidis typically causes gastroenteritis and is responsible for a global epidemic linked to poultry and egg production. Over the recent decades, *S.* Enteritidis has become a leading cause of invasive nontyphoidal *Salmonella* (iNTS) disease in sub-Saharan Africa (1, 2), and novel clades of this serovar have been isolated from individuals with bloodstream infection (3). In contrast to the Global Epidemic clade, these novel African clades are associated with high mortality in immunocompromised individuals and are typically multidrug resistant (MDR), representing an important public health challenge (4).

We used long-read sequencing to investigate the genome sequences of two representative *S.* Enteritidis strains of the Central/Eastern African clade (CP255 and D7795) and two of the Global Epidemic clade (P125109 and A1636). CP255 was isolated in the Democratic Republic of Congo (then Zaire) in 1991, from the blood of a child at the Institut Médical Evangélique, Kimpese, and is phenotypically MDR (amoxicillin, tetracycline, chloramphenicol, and streptomycin resistant) (5, 6). D7795 (pediatric patient/MDR) and A1636 (adult patient/fully susceptible) were isolated in 1998 and 2000, respectively, in Blantyre, Malawi (3). *S.* Enteritidis P125109, a UK PT4 isolate from 1988, was used as a reference (7–10).

A single colony of each isolate was grown for 16 h in 5 ml of Lennox medium at 37°C. Total DNA was extracted using the Quick-DNA universal kit (Zymo; catalog number D4069). DNA integrity was verified by 0.5% agarose gel electrophoresis at 90 V for 1.5 h. DNA purity/concentration were measured with a DeNovix DS-11FX spectrophotometer/fluorometer and Qubit double-stranded DNA (dsDNA) high-sensitivity (HS) assay kit. Long-read sequencing was performed by the Centre for Genomic Research (University of Liverpool, UK) in a PacBio single-molecule real-time (SMRT) cell (P6/C4 chemistry) using SMRTbell Template v1.0 (Pacific Biosciences; catalog number 100-259-100) library preparation with g-TUBE (Covaris) fragmentation and size selection of 15 to 50 kb with 0.75% agarose cassette (BluePippin; catalog number BMF7510). Illumina HiSeq sequencing was performed as part of the 10KSG project (11) and by MicrobesNG (UK) using the Nextera XT library prep kit (Illumina, USA) with modifications (2 ng DNA and 1 min PCR elongation) and 250-bp paired-end protocol. The reads were adapter trimmed using Trimmomatic v0.30, with a sliding window quality cutoff of Q15 (12).

Using Filtlong v0.2.0 (https://github.com/rrwick/Filtlong) and Illumina reads as a reference, we selected a subset of raw PacBio reads with the best quality and length to yield an approximate 100× coverage for each genome sequence. Selected long and short reads were assembled using Unicycler v0.4.4 in hybrid mode (13). The genome sequences were initially annotated using Prokka v1.13.7 (14) through Bacpipe v0.6 (https://github.com/apredeus/multi-bacpipe), automatically reannotated by GenBank with PGAP (15), and rotated to the origin at the *thrLABC* operon. Variant calling was done with Snippy v4.3.6 (https://github.com/tseemann/snippy) in contig mode.

Genome comparison revealed genomic degradation and differences in accessory genomes (Table 1). P125109 and A1636 carried the virulence plasmid pSENV, whereas D7795 carried pSEN-BT (3) and CP255 carried pSEN-DRC, which both had a pSENV backbone with multidrug resistance-encoding genes. Other plasmids were identified in A1636, D7795, and CP255. The prophage repertoire of A1636 resembled P125109 (7). Both D7795 and CP255 lacked ΦSE20 and instead carried P88-like and Fels2-like prophages.

**TABLE 1:** Characteristics and accession numbers of the genome sequences of four *S.* Enteritidis isolates

Isolate	Yr	GenBank accession no.	SRA accession no. for:		No. of Illumina reads (2 × 250 bp)	Illumina coverage (×)	No. of PacBio reads	PacBio read *N*_50_ (bp)	G+C content (%)	Genome size (bp)	Total no. of genes	Data for virulence plasmid:		Data for other plasmids:		No. of SNPs for isolate:^*b*^	
			Illumina reads	PacBio reads								Name^*a*^	Size (kb)	Name^*a*^	Size (kb)	P125109	D7795
CP255	1991	GCA_015240995.1	SRR12953596	SRR12953597	296,730	30.6	40,000	20,451	52.30	4,840,946	4,729	pSEN-DRC	96	pRSF1010 (16)	8.7	972	61
D7795	2000	GCA_015240855.1	SRR12953602	SRR12953603	813,469	83.5	103,762	16,718	52.30	4,869,504	4,777	pSEN-BT (3)	116	pRGI00316 (17)	4.9	1,017	0
A1636	1998	GCA_015241115.1	SRR12953598	SRR12953599	3,859,080	406.4	97,435	16,873	52.20	4,748,456	4,608	pSENV	59	pSE-GC	3.2	46	1,022
P125109	1988	GCA_015240635.1	SRR12953600	SRR12953601	587,289	61.9	92,761	17,272	52.20	4,745,224	4,606	pSENV	59			0	1,022

a Numbers in parentheses indicate references.

b SNPs, single nucleotide polymorphisms identified in whole-genome-based sequence comparison against either *S.* Enteritidis P125109 or D7795.

### Data availability.

The annotated complete genome assemblies of *S.* Enteritidis CP255, D7795, A1636, and P125109 have been deposited at NCBI GenBank. The BioProject accession number is PRJNA671837, and the individual BioSample accession numbers are SAMN16552338 (A1636), SAMN16552337 (CP255), SAMN16552336 (D7795), and SAMN16552335 (P125109). The Bacpipe annotation pipeline can be accessed at https://github.com/apredeus/multi-bacpipe.
